# The Therapeutics of Periodical Cephalalgia

**Published:** 1877-05

**Authors:** F. Bradnack

**Affiliations:** New York City


### BUFFALO

### Medical and Surgical Journal.

VOL XVI. MAY, 1877. No. 10.

### Original Communications.

:o:

ART. I.—*The Therapeutics of Periodical Cephalalgia.* By F. BRADNACK, M. D., New York City.

Written for the Buffalo Medical Journal.

Some time ago I contributed to the columns of this JOURNAL certain suggestions, (the result of my own experimentation), relative to the treatment of that *opprobrium medici*, periodical headache. Since that writing, further experience has enabled me, while adhering in the main to the plan then suggested, to add thereto, and also to suggest certain modifications of treatment in a class of cases which present numerous variations from the stereotyped symptoms of the disorder.

The object of this paper will be to formulate the therapeutical conclusions to which my additional experience has led me.

Prof. Austin Flint classifies periodical headache (cephalalgia) among the *opprobia medici*. Now, for a long time the disease has certainly belonged in that nosological category. But, I suspect that shortly it will belong there no more.

In my experience, the malady furnishes proof of the truth of Dr. Maudsly's apothegm, that, in no class of diseases are we enabled to produce, with proper scientific medication, such satisfactory therapeutic results as in diseases of the nervous system.

In my former paper I ventured the assertion that in most cases of periodical headache, cerebral congestion acts the part of a prominent pathological factor. My further experience substantiates the truth of this theory; and, I am confident that ophthalmoscopic examination will be found in a large number of cases to confirm it. Periodical headache is a disease of civilization. It is, so to speak, one of the penalties of civilization. Those who use their brains most are they who are most subject to this malady. In many cases its origin may be traced to excessive brain work.

I find that for practical purposes periodical headache is divisible into three distinct varieties. Each variety demands a different plan of treatment; and the treatment adapted to any one form will generally prove inoperative in the other two. For therapeutical purposes, these three varieties may be defined as:

1. Cephalalgia, due chiefly to functional gastro-hepatic derangement.
2. The so-called "sick headache."
3. Cephalalgia, due to encephalic engorgement.

In the first variety we find both gastric and hepatic disorder coexisting with *lithæmia* (to use Dr. Murchison's definition). The urine is periodically loaded with lithates, there are indications of hepatic torpor, resulting in the retention in the blood of cholesterine (the *cholesteræmia* of Flint). In these cases there exists some sympathetic cephalic congestion, but in a far less marked degree than in the other varieties. With this class of patients I find the following plan of treatment very effectual.

I first administer for three consecutive nights, at bed-time, one of the following pills:

| | | |
|---|---|---|
| ℞ | Mass. hydrarg. | |
| | P. aloes socot. | |
| | Ext. coloc. co. a a | gr. x. |
| | Ext. Jalapæ, | gr. j. |
| | P. Ipecac, | gr. iii. |

Ft. pil. No. vj. M.

I next give each morning before breakfast a wineglassful of the following mixture :

| | | |
|---|---|---|
| ℞ | Magnesii sulphat. | ℥j. |
| | Potass. bitart. | ʒj. |
| | Ferri. sulphat. | gr. xiii. |
| | Aquæ pur. | O ij. |

Ft. mixt. M.

The patient takes this upon getting out of bed, after which I direct him to suck the juice of an orange (athecerber the better) and then partake of a light breakfast, from which meal oleaginous and sacharine matters are mostly excluded. Dr. Murchison asseverates that in persons with lithatic urinary deposits, oleaginous and saccharine matters almost always disagree. My clinical experience has over and over again convinced me of the truth of this dictum. Therefore, I endeavor to reduce in this class of patients oleaginous and saccharine articles of diet to a minimum, especially the former. I give nothing to specially affect the encephalon or the spinal system, until after the alimentary canal has been thoroughly purged, and the morbid hepatic conditions ameliorated ; and, it has been my constant experience that nervine medicaments in this class of cases, fail if given prior to the thorough swabbing out of the *primœ viœ*, and the readjustment of the functions of the liver. But, after the alimentary canal and the liver have approximated their normal conditions, I find the following mixture very potent in first aborting the threatened attacks of cephalalgia, and, second, in greatly diminishing their frequeucy in such cases are not entirely cured. But 90 cases in 100 I deem curable by this method, providing there exist no other serious neural or visceral complication. The possibility of any continued persistent cephalalgia being due to syphilitic intracranial gummata must not be overlooked. However, usually syphilis is not concerned in the etiology of the affection now under consideration. I direct the following mixture to be taken twice daily for several weeks, and double doses to be taken whenever the cephalalgic prodromata are experienced :

| | | | |
|---|---|---|---|
| ℞ | Acidi nitro-muriatici dil. | | f ℥ss. |
| | Aquœ destel. | aa. | f. ℥ vi. |

S. f. ʒi. t. i. d. M.

In most of these cases the bromides, (which are so useful in another class, hereafter to be described) I find not only useless, but often injurious. Alkalies seem to be antagonistic here, while acids, acescent drinks and fruits (as oranges, lemons, Rhine wine), prove very valuable remedies. I find, also, that these patients are mostly injured by alcoholic drinks, especially malt liquors, ale and beer seeming almost invariably to derange both the liver and stomach. Dry wines and whisky are far less injurious to them, but they are mostly best without alcohol in any shape. On the other hand, a cup of clear coffee (the *cafe noir* of French breakfasts), does them great good. I direct them to use no milk, and as little sugar as possible, for milk in this form of hepatic trouble I find to be usually injurious. Without pretending to give an account of the *modus operandi* of the coffee, I suspect it acts as a gentle cholagogue ; for patients often report to me that the morning intestinal dejections present indications of the presence of bile. They also assert that they experience relief after the coffee, their heads feeling clearer, their senses brighter, and their general condition better. Thus it would appear as if the *fons et origo* of the morbific material in this class of cases were the liver. But, the chief object of this paper being therapeutical rather than etiological, I shall go no further into the causation of the pathological conditions than shall be requisite for clearness in diagnosis. I have no doubt that the non-elimination of cholesterine (due to hepatic inactivity), is the chief pathological condition in this variety of headache. The so-called bilious symptoms, the hebetude, the metalic taste in the mouth, the constipation, also the promptly remedial effects of the above plan of treatment, point, I think, clearly to the liver as, at least, the partial seat of the trouble. In these cases the bromides do no good. Contrariwise, they often do harm. The cephalic congestion which they so well combat in other cases, is here relieved by the continual moderate purgation. I find that these patients greatly suffer from a deprivation of fresh air ; and, indeed, I am compelled to regard this as one of the principal ancillary conditions in all the forms of periodical headache in this climate. The patients shut themselves up too much in ill-ventilated rooms, street-cars, ferry-boats and such like. They labor under a chronic insufficiency of oxygen, especially during the winter months. I advise the male patients suffering from this malady to ride on the *outside* of street-cars and ferry-boats, even in bad weather, telling them they had better run the risk of an occasional cold than undergo chronic poisoning by the inhalation of the mephitic air of the cars and ferry-boats in winter.

Persistence in this treatment for several weeks, avoidance of an excess of sacharine and oleaginous diet, plenty of fresh air, and attention to general hygiene, usually cures these cases. The cathartic pills are not repeated except upon a recurrence of the cephalalgic prodromic symptoms, when I administer one or two for three consecutive nights.

The second variety of P. cephalalgia is that popularly described as "sick headache." This appears to be a sort of paroxysmal cephalic discharge, in the same way in which epilepsy may be so defined. As it were, morbific explosive material accumulates in the system during three or four weeks; and when a certain amount has become stored up, an explosion takes place. Though not a very scientific, this seems to be a practical description of the phenomena manifested in this malady. These attacks appear to be, as it were, crises in the system, similar to thunder storms in nature. There is dull, heavy, depressing weather before the electric storm, succeeded by clear air, bright skies and fair weather. In the human system previous to the outburst, there are present languor, debility, hebetude, anorexia and general malaise. After a continuation of these symptoms for two or three days, the explosion (so to speak) occurs, and, after a stormy period, nature returns to her normal condition. Now, this state of things is very dissimilar from that which exists in the hepatic form of this disease, although in some instances there may be vomiting of bile. Prophylactic treatment may be advantageously employed in this form. Between the paroxysms I prescribe the following:

| | | |
|---|---|---|
| ℞ | Tinct. Rhei | f ℥ iii. |
| | Sodæ bicarb. | ʒ j. |
| | Gaulth. spts. | f ʒ ij. |
| | Sirupi Simp., | f ℥ iii. |

S. f ʒ j. t. i. d. M. f. B.

With this I give daily very small doses of the sulphate of magnesium, a few grains merely. And, in this connection, it has seemed to me that full justice has never been done to the *cholagogue* powers of the sulphate of magnesium. My attention was first called to the cholagogue properties of this salt, by an eminent member of the profession, Dr. Edward Howard, M. R. C. P., formerly of Lockport, N. Y. I have since had frequent opportunities of demonstrating the truth of his assertion, that the sulphate of magnesium is often quite as efficient a cholagogue as is mercury, possessing, moreover, the manifest advantage over the latter drug of never being attended by undesirable sequelæ. In this hepatic form of periodical headache I have repeatedly administered sulphate of magnesium in frequent minute doses with marked benefit to the chylo-poietic viscera. But, to obtain satisfactory results, the salt must be administered always in small frequent doses, and be continued during a lengthened period. Not its purgative, but its *alterative* qualities are here desired, using the word "alterative" in the true sense of that much slandered term.

In connection with the above, these patients take the following in some cases with benefit:

℞ Zinci oxidi, ℈ ii.

Ext. hyoscyami, ʒ i.

Ft. Pil. No. xvi. M.

S. 1 twice daily.

This treatment, if it do not abort, will often lessen the severity of the attack. During the paroxysm (waiting if possible until after the stomach has been evacuated by vomiting), I give 25 grains of bromide of potassium dissolved in a cup of strong black tea (English breakfast.) Between the paroxysms, in a certain proportion of cases, great benefit to the general health usually results from frequent small doses, (say M iii thrice daily) of tincture of nux vomica. This variety of headache, though very persistent, will frequently yield to the above plan of treatment.

The third variety of this malady is a *neurosis* pure and simple. It is important that the physician should recognize this fact. Complicated with this cephalalgia there is neither gastric nor hepatic derangement, nor is there visceral disorder of any kind. The morbid conditions are located solely in the cerebro-spinal axis. Neither does it originate, as does the hepatic form, in peripheral irritation, but centrically. It is, if I may so say, an ideal neurosis. The victims of this form of headache are usually intellectual persons. In them the brain is largely developed. By them it is constantly, and often, excessively used. The following constitutes a description of a typical case of this form of cephalalgia. After several days of severe brain work, perhaps, or after unusual emotional excitement, or mental worry, there ensues a brief period of general malaise. Then commences steady, throbbing, increasing pain in the head, most intense in the cerebrum. The whole head is exceedingly hot, the sensation of heat being greatest over the lower portion of the cerebellum and the medulla oblongata. Muscæ volitantes float before the eyes. There is manifest congestion of the external visible parts of the head (significant of a similar condition intracranially). The conjunctivæ are congested; the temporal arteries pulsate visibly ; the cheeks are scarlet. Occasionally nature opens a safety valve in the shape of epistaxis, thereby relieving the tension of the encephalic arteries. There is jactitation, with twitching of the limbs, and, quite often, in the upper extremities, a sensation of formication. Superadded to these, there is sometimes vertigo, and also the gait, if walking be attempted, may be titubating. The eyes are unusually bright. Vision is unaffected, except by the muscœ volitantes. Audition is sometimes affected. There may be tinnitus aurium. There may also be experienced the sensation of a continuous buzzing in the ears. There is violent pain in the head which becomes at times excruciating. In some instances there is felt the sensation of tingling in one arm or leg ; at other times a numbness of the same extremities, these symptoms being manifestly due to encephalic congestion. The extremities are invariably cold. The head and cervical regions are burning hot. These last two symptoms are always present, and are almost pathognomonic. But with all these evidences of cephalic disturbance, there is neither gastric nor hepatic disorder. In many cases even the appetite is unaffected. Now, it appears to me that this malady is as much entitled to be denominated a neurosis, as is trifacial neuralgia. The paroxysm may last 12, 24, or 48 hours.' It seldom continues longer. The following treatment is that which I have found useful during the paroxysm. First, place the feet and hands in mustardized hot baths, the water being as hot as can be borne. Next. apply an ice-bag to the skull and over the nucha, and administer the following mixture at once :

| | | |
|---|---|---|
| ℞ Potassii bromidi, | | gr. xxx. |
| Tinct. lupuli, | | f ʒ iii. |
| Aq. menthœ pip. | aa. | f ℥ iv. |
| | | M. |

After this direct the patient to recumb in an easy chair, and not in bed or upon a lounge. The reason for this is, that the horizontal position facilitates the gravitation of blood into the skull, and is therefore injurious. After the baths hot bricks to the hands and feet are usually found grateful and helpful. Frequently a slight meal of dry toast and weak black tea (milkless and sugarless), acts as an important adjuvant to the medical treatment, probably as a derivative, drawing off some blood from the engorged encephalic vessels to the stomach. Anyhow, whatever may be its *rationale*, it is often a useful ancillary measure, and is worthy a trial in every case. During sleep the head should be elevated by at least three pillows to counteract the effcts of gravitation. This point is important, inasmuch as a great part of the trouble in this case is due to actual mechanical pressure of the blood upon the contents of the skull. In fact, the essential pathological condition is intracranial vascular engorgement. This fact should never be lost sight of. Most of the morbid symptoms are the result of hæmal pressure. Therefore, the upright or nearly upright position is absolutely essential in this variety of cephalalgia ; while in the hepatic form it is of but secondary importance. Upon the same principle, any clothing which constricts the neck or chest is injurious. But the feelings of the patient usually compel him willy-nilly, to disbarrass his chest and neck of anything which constricts those parts. Massage of the lower extremities will be found useful in diverting blood from the engorged encephalon. In this variety of periodical headache, neither cathartics, cholagogues, nor acids are indicated. The one sole indication is to decongest the encephalon. This indication we are enabled to meet satisfactorily by means of those invaluable remedies, the bromides. After five years' experience of their use in a large number of cephalalgic cases, I do not hesitate to declare my conviction that in encephalic vascular engorgement the bromides are as much entitled to the name of specific as is cinchona to the name of specfiic in intermittent fever, or as is iodide of potassium in secondary syphylis. In 99 cases in 100 the bromides will reduce the cranial congestion during the paroxysm. I combine the lupuline with bromide of potassium from an experience of its usefulness in fulfilling the same indication. My friend Dr. W. C. Earl, of Buffalo has reported to me several successful cases of this form of cephalalgia treated after the above manner.

The proof of the correctness of diagnosis in these cases lies in the fact that upon the subsidence of the encephalic engorgement, the multitudinous aforementioned symptoms vanish as if by magic. Indeed, to say the truth, I doubt if the astrologists and alchemists of the dark ages ever possessed an elixir more potent against mortal pain than is a solution of the bromide of potassium in these cases. In this form of headache the effect of this drug may justly be described as magical. I have in some instances substituted bromide of lithium for bromide of potassium, but I am not aware that it possesses any marked advantage over the potassium salt, while it has the disadvantage of being much more expensive. Hence I seldom now use it in these cases. So much for the treatment during the fit.

I next come to speak of the treatment between the paroxysms, which forms by far the most important part of our duty in these cases. This congestive form of periodical headache is the most frequent of the three varieties. I think it will be found that in most of these cases there has been either excessive brain work, or excessive emotional disturbance, or both. The eccentric but sapient Dr. Abernethy asseverated that the two great killing powers in this world are "stuff and fret." I believe that in the hepatic form of periodical headache "stuff" (*i. e.*, over-eating of improper food) is often the cause of the trouble, and am equally sure that in the congestive form "fret," either alone or combined with excessive brain work is responsible for the pathological conditions therewith met. Therefore, the first thing to be done is to insure as much as possible *rest of the brain* ;—not cessation of mental work, but diminution thereof. There exists in these cases, doubtless, a derangement of the cerebral vaso-motor system, in consequence of which there ensues more or less chronic congestion of the brain, and a tendency to periodical engorgements thereof is established. Therefore the treatment has a double object—first, the reduction of this chronic abnormal increase in the diameter of the cephalic blood-vessels ; second, the prevention of the (so to speak) periodical explosions. And, in truth, the word "explosion" but little exaggerates the condition ; for, unquestionably, if there existed in one of the cerebral arteries a weak spot, the artery would be very apt to give way at that spot under the hæmal pressure to which it is subjected during these attacks. In other words, apoplexy would occur. Therefore, this variety of periodical headache must, in consideration of this danger, be regarded as much the gravest of the three ; and the possibility of apoplexy during an attack should not be overlooked by the practitioner. To modify this abnormal condition of the vaso-motor system, I find the following mixture admirably adapted :—

| | | |
|---|---|---|
| ℞ | Potassii bromidi, | ℥ j. |
| | Sodii bromidi, | ʒ ij. |
| | Potassii iodidi, | gr. vij. |
| | Ammon. bromidi, | ʒ j. |
| | Aq. menthœ pip. | f ℥ viij. |

S. fʒj. in aqua, t. i. d. M.

I may be accused of polypharmacy (a frightful accusation at best,) in this prescription, but so satisfied am I of its efficacy that I shall not mind *that*. I have also found in several recent cases that tincture of hop does good in two ways ; first, it acts as a slight cephalic decongester, and, second, as a wholesome sedative to the nervous system. In a case recently under my observation, I suggested to the patient, who was in the habit of smoking tobacco to excess, to substitute hops for tobacco in his pipe. He has done so, and reports very great benefit from this fumiferous succedaneum. He does not find it necessary altogether to eschew tobacco, but substitutes hop for one-half of his daily allowance. This patient, prior to my treatment, had suffered for months such persistent engorgement of the cerebellar and upper spinal regions that he found it impossible to sleep without a cold wet towel applied to the occipital and cervical regions. So soon as these parts became heated by contact with the pillow he experienced bad dreams, uneasy sensations, followed by waking. I suggested that he should smoke a pipe of hops immediately before going to sleep, and also recommended a tightly-packed hop-pillow instead of the usual feather pillow. The patient reports great benefit and comfort from these measures, and tells me that he is now rarely troubled by the cerebellar heat, or by bad dreams. Instead of waking a dozen times each night, he now sleeps unbrokenly until morning. In some few of these cases both coffee and tea disagree ; but, in most of them these beverages do no harm, so long as they are not used to excess. The same remark applies to the use of tobacco. Unless there be a marked idiosyncrasy in respect of tobacco, I do not find a small quantity daily, injurious. But, the case is different with the stronger forms of alcoholic drinks. These should be tabooed by the physician. Indeed I would inhibit all alcoholic potations with the single exception of lager beer, which is frequently positively beneficial by reason of its hypnotic qualities. On this account I frequently utilize it as a vehicle, the nocturnal medicine to be mixt in a tumbler of it. As a hypnotic, lager beer certainly rivals the much-lauded Tarragona wine, and has the advantage over it of being vastly cheaper.

Sedentary occupations favor the congestive form of periodic headache, especially when severe intellectual labor is conjoined thereto. To counteract this, I recommend these patients to walk as much as possible in the open air, the muscnlar exercise tending to draw off the hœmal surplusage from the brain. Friction of the extremities, shampooing the skin, and massage act in a similar manner, and are proportionately beneficial. Bearing in mind that the condition we have here to deal with is simple hyperœmia, it becomes manifest that any method which tends to attract blood from the engorged encephalon will be useful in the direct ratio of its activity. And in this connection I have no hesitation in avowing my conviction that clinical facts in some of these cases seem to me to clearly indicate blood-letting, quite as clearly as do certain cases of apoplexy; for, manifestly, it is better by a timely venesection to prevent the occurrence of apoplexy (the risk of which in these cases is by no means inconsiderable) than to attempt by the same process to ameliorate the condition after cerebral hœmorrhage has taken place. That apoplexy is threatened in some of these cases, there can be no reasonable doubt. The numbness, tingling, stuttering speech, the facial and retinal congestion all point clearly to this danger.

I find that all alkaloids and forms of cinchona do mischief to these patients. Clinical facts furnish, to my mind, convincing proof that cinchona produces cephalic hyperœmia, and not the reverse, as has lately been asserted by several authorities. It being necessary in some instances to give roborant medicines for the purpose of counteracting the debilitating effects of the bromides (which effects, however, in most cases are slight) I have sometimes administered tincture of cinchona and sulphate of quinia, but have usually been compelled to discontinue them, and to substitute tincture of chiretta or nux vômica, both of which drugs act excellently well as tonics in these cases. Sometimes the bromides and zinc oxide in alternate doses do more good than does either drug separately. No absolute rule can be laid down in these anomalous cases; the practitioner is necessitated to proceed tentatively. But, in the great majority of cases of this variety, I am confident that the above plan of treatment will be found successful, so successful as to be almost entitled to the name of specific; and, in so saying, I am fully aware how few remedies or plans of treatment can justly lay claim to that title. The plan above sketched for treatment during the paroxysms is intended to be palliative. It scarce ever fails to prove so. The treatment between the paroxysms is intended to be curative, and proves so in a large majority of cases.

In conclusion, it is proper to call attention to the importance of correctly diagnosticating the particular variety of headache under which the patient may labor, inasmuch as the treatment of one variety is inoperative if not injurious in the two others. The differential diagnosis presents no special difficulty. The history of the prodromata is usually sufficient to make each case plain; and, in the true congestive form the visible engorgement of the facial vessels is in itself pathognomonic.

In consideration of the great suffering caused by periodical headache, and also of the fact that the text-books say nothing on the subject, it has seemed to me desirable to formulate my experience in the treatment of this truly distressing malady, in the hope of transferring it from the category of *opprobria medici,* and placing it in that of diseases which are almost certainly amenable to scientific treatment. And, in truth, it seems to me it should to every true physician, be a source both of pride and of gratulation that a painful disease which has hitherto been deemed well-nigh incurable, should, by the progress of our divine art, come to be regarded as one of the most curable of human maladies. Such a result constitutes one of those triumphs which go far towards making amends for many of those vexations and grievances to which the life of the conscientious physician is so peculiarly subject.

